# Irreversible cardiotoxicity induced by trastuzumab: a systematic review based on a pharmacovigilance case report

**DOI:** 10.47487/apcyccv.v6i1.456

**Published:** 2025-02-12

**Authors:** Víctor E. Lechuga-Noa, L. Yesenia Rodríguez-Tanta, Tania del Pilar Solis-Yucra, Efraín Cesar Rojo Rosales

**Affiliations:** 1 Instituto Nacional Cardiovascular - INCOR- EsSalud, Lima, Perú. Instituto Nacional Cardiovascular - INCOR- EsSalud Lima Perú; 2 Grupo Peruano de Investigación en Medicamentos, Políticas y Servicios farmacéuticos. Universidad Científica del Sur, Lima, Perú. Universidad Científica del Sur Grupo Peruano de Investigación en Medicamentos, Políticas y Servicios farmacéuticos Universidad Científica del Sur Lima Peru; 3 Hospital Nacional Edgardo Rebagliati Martins - EsSalud, Lima, Perú. Hospital Nacional Edgardo Rebagliati Martins - EsSalud Lima Perú

**Keywords:** Breast Neoplasms, Cardiotoxicity, Trastuzumab, Pharmacovigilance, Neoplasias de la Mama, Cardiotoxicidad, Trastuzumab, Farmacovigilancia

## Abstract

Irreversible cardiotoxicity (IC) induced by trastuzumab (TZB) is a rare but serious adverse event. As a result, its characteristics and the specific factors related to exposure remain poorly understood. This study aims to synthesize and evaluate the existing evidence on IC. We presented a pharmacovigilance case of long-term IC and conducted a systematic review (SR) of the clinical manifestations of cases reported worldwide. We reported the case using the CARE guidelines checklist and assessed the causality using the modified Algorithm of Karch and Lasagna. Following PRISMA guidelines, we conducted the SR using defined terms in PubMed, Embase, Scopus, and Web of Science from inception until June 2023. This SR included five case reports, including the pharmacovigilance case reported by us. While patients exhibited different severe clinical characteristics, receiving TZB at a 6 mg/kg dose was consistent. Despite varying treatment durations, the median time of IC diagnosis was 10 months, and the average difference between the basal and the final left ventricular ejection fraction was roughly 30%. According to the modified Karch and Lasagna algorithm, all cases were ranged from possible to probable. While TZB is generally considered a reversible cardiotoxic antineoplastic, clinicians and regulators must be aware of the potential IC risk with long-term manifestations. Vigilant cardiac monitoring and further research are crucial to better understanding and managing this serious adverse event.

## Abstract (es)

La cardiotoxicidad irreversible (CI) inducida por trastuzumab (TZB) es un evento adverso poco frecuente pero grave. Como resultado, sus características y los factores específicos relacionados con la exposición permanecen poco comprendidos. Este estudio tiene como objetivo sintetizar y evaluar la evidencia existente sobre la CI. Presentamos un caso de farmacovigilancia de CI a largo plazo y realizamos una revisión sistemática (RS) de las manifestaciones clínicas de casos reportados a nivel mundial. Reportamos el caso utilizando la lista de verificación de la guía CARE y evaluamos la causalidad empleando el algoritmo de Karch y Lasagna modificado. Siguiendo las directrices PRISMA, llevamos a cabo la RS usando términos definidos en PubMed, Embase, Scopus y Web of Science desde su inicio hasta junio de 2023. Esta RS incluyó cinco informes de casos, incluido el caso de farmacovigilancia identificado en nuestro hospital. Aunque los pacientes presentaron diferentes características clínicas graves, el uso de TZB a una dosis de 6 mg/kg fue consistente. A pesar de la variabilidad en la duración del tratamiento, la mediana del tiempo de diagnóstico de la CI fue de 10 meses, y la diferencia promedio entre la fracción de eyección del ventrículo izquierdo basal y final fue aproximadamente del 30%. Según el algoritmo modificado de Karch y Lasagna, todos los casos se consideraron con una causalidad entre posible y probable. Aunque generalmente se considera a TZB como un antineoplásico con cardiotoxicidad reversible, los clínicos y reguladores deben ser conscientes del riesgo potencial de CI con manifestaciones a largo plazo. Es fundamental un monitoreo cardíaco riguroso y realizar más investigaciones para comprender y manejar mejor este evento adverso grave.

## Introduction

Breast cancer (BC) is the most common cancer among women worldwide. ^1^^)^ About 20% of BC cases exhibit an overexpression of the human epidermal growth factor receptor 2 (HER2), linked to a poor prognosis. ^2^^)^ Trastuzumab (TZB), a monoclonal antibody, has significantly improved overall survival in BC patients with HER2 overexpression. However, many patients develop trastuzumab-induced cardiotoxicity, a cancer treatment-related cardiac dysfunction (CRTCD) ranging from asymptomatic left ventricular ejection fraction (LVEF) decline (which can drop below 40% in severe cases) to mild dysfunction not requiring treatment intensification or severe dysfunction requiring inotropic support, mechanical circulatory assistance, or heart transplant evaluation. ^3^ This form of CRTCD may be reversible and asymptomatic upon discontinuation of TZB, but in some cases, it may progress to irreversible cardiotoxicity. ^4^

Irreversible cardiotoxicity (IC) refers to permanent damage and loss of cardiac myocytes, leading to a significantly higher risk of heart failure and related complications. This severe condition is well-documented following the administration of anthracyclines and alkylating agents, and to a lesser extent with TZB. ^5^^)^ While clinical trials have demonstrated TZB’s efficacy and safety, their relatively short follow-up periods and strict eligibility criteria may limit the identification of long-term, irreversible cardiac effects. ^6^ Pharmacovigilance data play a crucial role in detecting these late-onset events, complementing pre-approval clinical trials, and providing real-world insights into the cardiac safety of TZB.

Despite scientific literature reporting that TZB may affect cardiomyocyte function without causing cell death, patients who are also receiving anthracyclines or have a higher risk of heart problems may be at an increased risk of developing IC. Therefore, early trastuzumab-induced cardiotoxicity detection is crucial to prevent its progression to irreversible cardiotoxicity. It is recommended that patients receiving TZB undergo echocardiographic screening every three months during treatment, regardless of their baseline risk, to detect subclinical and asymptomatic cardiotoxicity.^7^^)^

Here, we report a case of a postmenopausal woman with HER2+ BC who developed long-term irreversible cardiotoxicity despite discontinuing TZB and initiating cardioprotective treatment. After her twelfth cycle of TZB, she was diagnosed with pneumonia; however, her condition progressed following TZB rechallenge, suggesting an underlying cardiac involvement that was not initially recognized. Due to the limited data on irreversible cardiotoxicity associated with TZB, this case highlights the importance of conducting a systematic review to compile existing evidence, better understand risk factors, and enhance strategies for monitoring and prevention.

The present case report and systematic review aim to address key questions regarding the frequency, onset time, and risk factors of this adverse event. Additionally, it seeks to synthesize and evaluate the existing evidence on irreversible cardiotoxicity associated with trastuzumab from a pharmacovigilance perspective.

## Materials and methods

After obtaining the patient’s consent, we collected and assessed clinical information of IC due to TZB. The report was prepared following the CARE guidelines checklist. Building on this pharmacovigilance case report, we conducted a systematic review (SR) to further investigate the association between IC and TZB. The SR was reported following the Preferred Reporting Items for Systematic Reviews and Meta-Analysis (PRISMA) guidelines. ^8^ The protocol, updates, and modifications were registered in the International Prospective Register of Systematic Reviews (PROSPERO) with the number CRD42022323046.

### Search strategy

We conducted a scientific literature search using a combination of controlled vocabulary and search terms from the inception until June 2023 in the following databases: 1) PubMed/Medline, 2) EMBASE, 3) Scopus, and 4) Web of Science. We adjusted the search terms to the requirements of each database, with no search filters applied. The search strategies for all databases are provided in the Supplemental File. We also reviewed the reference list of the selected studies for potentially eligible studies. Grey literature was not included in the search due to variability in quality and the lack of peer review, which could compromise the validity and reliability of the findings. Finally, we used EndNote X10 software to select the studies and eliminate duplicates.

### Eligibility criteria and selection of studies

We included only case reports and case series of irreversible cardiotoxicity potentially associated with TZB, manifested as irreversible heart failure or an asymptomatic LVEF decline, using 50% as the cutoff value. ^3^ The selected case reports described women with HER2+ BC treated with TZB with or without chemotherapy/radiotherapy. We also included hand-searched original studies listed in the retrieved articles that met the inclusion criteria.

Two independent reviewers (Y.R. and V.L.) implemented a two-stage process to select studies: title, abstract, and full-text review. Discrepancies were resolved by consensus between both reviewers.

### Data extraction

One reviewer (T.S.) extracted relevant data from eligible studies in a pre-designed extraction form in an Excel sheet, including location and period, patient’s characteristics (age, BMI, menopausal status, cardiovascular history, heart failure history, HER2+ BC stage), last cycle of TZB before cardiotoxicity, adjuvant treatment, dose, other therapeutic schemes used, anthracycline use, the interval between exposure to TZB and IC, basal LVEF and strain assessment, baseline and post-treatment troponin and N terminal pro-brain natriuretic peptide (NT-proBNP) levels, frequency of their use in the cardiac monitoring, IC definition, heart failure symptoms, and reversibility assessment. A second reviewer (E.R.) performed a quality control of the extraction process.

### Adverse drug reaction (ADR) causality assessment

We used the modified Karch and Lasagna algorithm to evaluate the causality of ADR in each reported case. The assessed domains of the modified Karch and Lasagna algorithm include 1) Temporal sequence, 2) Previous knowledge, 3) De-challenge, 4) Re-challenge, 5) Alternative causes for ADR, 6) Factors influencing the causality assessment, and 7) Complementary examinations.

### Data synthesis

Due to the high variability of information obtained from the included case reports, we conducted a descriptive analysis. Categorical variables are presented as frequencies and percentages, while continuous variables are reported as means with standard deviations for those that follow a normal distribution. We present medians and interquartile ranges (IQR) for non-normally distributed continuous variables. Statistical analysis was performed using Excel 2016.

### Ethical aspects

Institutional consent for publication was obtained from the patient, and the case has been presented with her signed consent. On the other hand, ethics approval is not required as this is a systematic review and a case report (secondary analysis).

### Risk of bias in individual studies

Two independent authors (Y.R. and V.L.) used the Murad *et al*. tool ^9^ to assess the quality of the case reports, incorporating the quality classification and the modification of six questions formulated in the article by Hosseini *et al*. ^10^^)^ Scores of 5-6, 4, and 0-3 were considered “good,” “fair,” and “poor” studies in terms of quality, respectively. Any disagreements were resolved through joint discussions and consensus between both reviewers.

## Results

### Case report

We report a case of IC possibly related to TZB as an indication for HER2+ breast cancer, with an initial manifestation of dyspnea. The patient is a 61-year-old Peruvian woman with a past medical history of hypertension managed with losartan over a decade; type 2 diabetes controlled with metformin, hypothyroidism, and obesity. She was diagnosed with stage 3 HER2+ BC in September 2017. Upon initial diagnosis of BC, she underwent breast-conserving surgery, and the immunohistochemical biopsy confirmed infiltrating ductal carcinoma. The markers Ki-67 and c-erbB2 were positive, while estrogen and progesterone receptors were negative.

The patient started with four cycles of a 3-week regimen of doxorubicin (60 mg/m^2^) and cyclophosphamide (600 mg/m^2^). Before starting chemotherapy, her LVEF was 68%. After completing four cycles, her LVEF decreased to 56%. Subsequently, she received adjuvant TZB weekly in combination with paclitaxel. The patient received a 4 mg/kg loading dose of TZB followed by a 2 mg/kg maintenance dose. No serious adverse events were manifested during the initial phase of the treatment.

Five minutes after the twelfth TZB infusion, the patient experienced shortness of breath, tachycardia (heart rate 130 bpm), high blood pressure (160/102 mmHg), SatO_2_ of 90%, a high respiratory rate of 34 bpm, and PaO_2_/FiO_2_ of 135. The multislice spiral computed tomography (CT) of the thorax revealed interstitial pneumonitis, and the electrocardiogram (ECG) indicated right ventricular hypertrophy. Laboratory data included NT-ProBNP (1750 pg/mL), fibrinogen (467.15 mg/dL), LDH (546 U/L), AST (546 U/L), ALT (502 U/L), alkaline phosphatase (345 U/L), and glucose (386 mg/dL). The patient was transferred to the Interventional Pulmonology Unit, where she was diagnosed with community-acquired pneumonia with a normal white blood cell count and was treated with IV piperacillin/tazobactam for seven days.

Two weeks later, the patient received the thirteenth cycle of adjuvant TZB and radiotherapy (RT) at 400 cGy/2 fractions. The following week, the patient again experienced shortness of breath. The ECG revealed a left bundle branch block, and a transthoracic echocardiogram (TTE) showed an LVEF of 37%, indicating heart failure. Consequently, she began treatment with bisoprolol and spironolactone. A follow-up TTE performed 15 days after the thirteenth cycle of TZB showed an LVEF of 35% and diastolic dysfunction. This led to the discontinuation of TZB, although she continued with RT.

**Figure 1:**
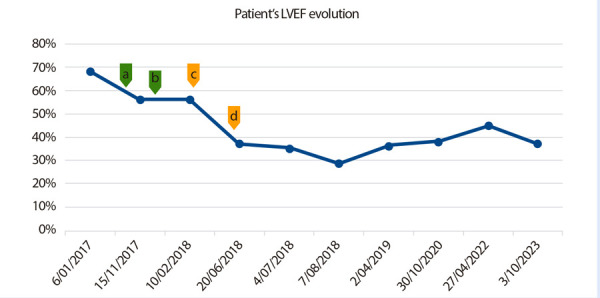
Patient’s LVEF evolution

Two months later, a follow-up TTE revealed an LVEF of 29% and diastolic dysfunction. Despite receiving pharmacological treatment and discontinuing TZB, her ejection fraction remained below normal limits through four subsequent evaluations until 2023 (Figure 1). She was diagnosed with long-term irreversible cardiotoxicity. Based on the modified Karch-Lasagna algorithm, the causal relationship between IC and TZB was categorized as “probable” with a score of 7 out of 12 points. The absence of basal cardiac biomarkers and echocardiographic strain data, along with prior doxorubicin use, were considered limitations in the assessment.

a=October 2017: First doxorubicin cycle; b= January 2018: Last doxorubicin cycle; c= February 2018: First trastuzumab cycle; d= June 2018: Last trastuzumab cycle

It is important to note that despite this setback, the patient showed clinical improvement during oncology check-ups from 2018 to 2023. Laboratory results consistently indicated stable cardiac biomarkers, with normal levels of NT-proBNP and troponin and negative tumor markers (CEA and CA 15-3). Furthermore, other imaging tests did not reveal any malignant lesions.

### Systematic review search results

**Figure 2:**
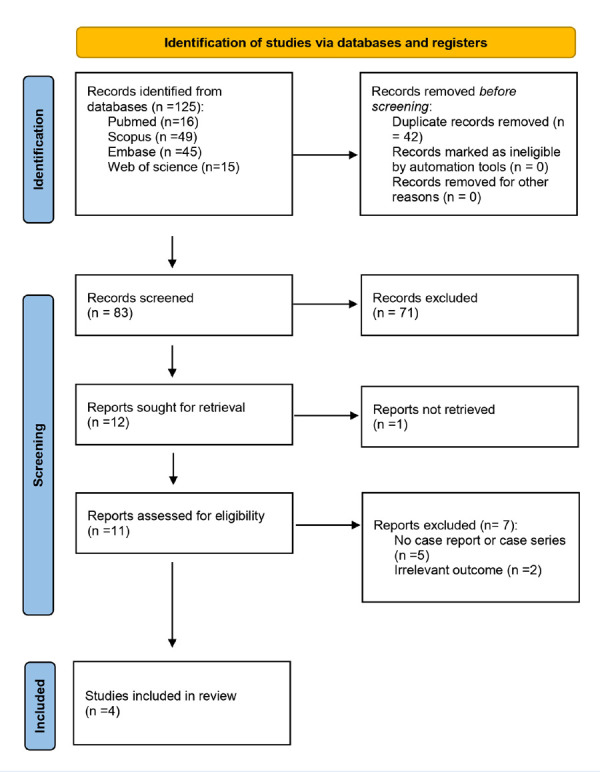
PRISMA Flow Diagram

We identified 125 individual records through our search. After removing 42 duplicates, we screened 83 records based on title and abstract. We assessed 11 studies in full text and included four cases of patients with IC potentially attributable to TZB. The PRISMA 2020 flow chart summarizing the screening process is in Figure 2. ^11^^-^^14^

Our analysis included five case reports, four of which were from the systematic review and one from our hospital. Patients were from five different countries (Japan, Italy, Russia, the USA, and Peru). The mean age was 52.6 (SD ±7.30). None of the patients had a prior history of heart failure, although one patient had hypertension and obesity. All patients had HER2+ BC, ranging from stage III to IV. Only one patient experienced bilateral involvement. (Table 1)

**Table 1:** Study and Patient characteristics

Case ID	Country	Age	Obesity	Cardiovascular history	Heart failure history	Laterality	HER2+ breast cancer stage (stage AJCC-TNM)	Mastectomy	LVEF basal	LVEF Lowest value	NYHA class	LVEF screening	Strain assessment	baseline troponin level (ng/ml)	baseline proBNP level ( pg/mL)	post-treatment troponin level (ng/mL)	post-treatment proBNP levels (pg/mL)	Heart failure symptoms	LVEF last value	Cardioprotective medication ^a^
New case 2018	Peru	61	Y	Y	N	Right	Stage III	N	68%	29%	III	EcoCG	N	NI	NI	NI	May 2018: 1750	Y	37%	Y
Tanaka S. 2017	Japan	52	NI	N	N	Left	Stage IV (T4b N3c M1).	N	64 %	39 %	NI	EcoCG	N	NI	NI	NI	NI	N	41 %	N
Skopets I. 2015	Russia	46	NI	N	N	Left	Stage III (T3N3M0)	Y	NI	28%	III	EcoCG	N	NI	NI	NI ^b^	Agu 2014: 12512 Dec 2014: 7782	Y	33%	N
Bordin P 2015.	Italy	45	NI	N	N	Bilateral	Stage IIIA o IIIB (Right pT2N3aMx) Stage IA (Left pT1cN0Mx)	Y	55%	20%	NI	EcoCG	N	NI	NI	NI	NI	Y	20%	N
Tham Y.L. 2002	USA	59	Y	Y	N	Right	stage III (pT3N3Mx)	Y	57 %	24 %	III	MUGA	N	NI	NI	NI	NI	Y	24 %	N

LVEF= Left ventricular ejection fraction, NYHA class= New York Heart Association Functional Classification, Y= Yes, N= No, EcoCG = Echocardiography, NI = No information, MUGA = Multigated acquisition scan.

a In all cases, TZB treatment was stopped when there was a decrease in LVEF.

b It is mentioned that the patient has no alteration in troponin values

Three patients had previously received doxorubicin, while one patient had received breast irradiation either before or during the current treatment. Four patients received 6 mg/kg of TZB. A patient with stage IV breast cancer received paclitaxel and TZB, and those in stage III were also given anthracycline (doxorubicin). A postmenopausal woman received letrozole. The total duration of the chemotherapy ranged from 4 to 24 months. The median time from starting the TZB regimen to IC was 10 months (interquartile range: 7 - 21 months) (Table 2)

**Table 2:** Treatment characteristics

Case ID	Chemotherapy duration (months)	Treatment approach	Anthracycline Use	Interval between first exposure to TZB and cardiotoxicity (months)	Interval between first exposure to trastuzumab and lowest LEFV measurement (months)	Treatment description
New case 2018	10	Adjuvant	Previous	4	6	Oct 2017- Jan 2018: Doxorubicin 60 mg/m^2^ + Cyclophosphamide 600 mg/m^2^ (4 Cycles) Feb 2018- Jun 2018: Paclitaxel 90 mg/m^2^ + Trastuzumab loading dose 4 mg/kg, then 2 mg/kg
Tanaka S. 2017	24	NI	N	24	24	Sep 2012- Dec 2012 : Paclitaxel 90 mg/m^2^ + Trastuzumab 2 mg/kg every week (12 cycles) Dec 2012- Sep 2014: Trastuzumab 6 mg/kg alone every 3 weeks
Skopets I. 2015	21	Adjuvant	Previous	16	18	Nov 2012 - Feb 2013: Doxorubicin 60 mg/m^2^ (6 cycles) May 2013 - Jan 2014: Trastuzumab (dose not report) every month Feb 2014: Trastuzumab (dose not report) + Placlitaxel (6 cycles) Until July 2014: Traztuzumab alone (dose not report)
Bordin P 2015.	7	Adjuvant	Concomitant	4	8	Until Dec 2005 : Doxorubicin +Paclitaxel (dose not report) Jan 2006- Apr 2006: Letrozole 2.5 mg + Trastuzumab (loading dose 6 mg/kg, then 4 mg/kg every 3 weeks) Apr 2006: Letrozole alone 2.5 mg
Tham Y.L. 2002	4	Neoadjuvant	Concomitant	5	5	Trastuzumab loading dose 4 mg/kg, then 2 mg/kg weekly + Docletaxel 100 mg/m^2^ every 3 weeks (4 cycles)

NI = No information, N= No

Before initiating TZB therapy, all five patients had a baseline LVEF within the normal range as assessed by TTE, averaging 61% (SD ±6.06). However, following treatment, the average lowest LVEF recorded was 28% (SD ±7.11), leading to the development of symptomatic grade III heart failure according to the New York Heart Association (NYHA) classification. At the latest assessment, the average LVEF was 31.2% (SD ±8.98), representing a significant decline of approximately 30% from baseline to final evaluation (Table 1). Compared to our case, none of the other four cases were initially misdiagnosed; all were confirmed as IC from the onset of the symptoms. All cases discontinued TZB upon declining LVEF values, with our patient being the only one to receive medications for treating the cardiotoxicity.

Finally, we assessed the causal relationship between IC and TZB in each included case. Using the modified Karch and Lasagna algorithm for ADR causality assessment, three cases were classified as “possible,” one as “probable”, and our case was also classified as “probable” (Table 3). Three cases were judged to have “good quality” (Table 4).

**Table 3:** Karch and Lasagna assessment

Karch and Lasagna score evaluation									
Case ID	Temporal sequence	Previous knowledge	Drug withdrawal effect	Rechallenge effect	Alternative causes	Contributing factors	Additional information	Total score	Category of causality
New case 2018	2	2	2	0	-1	1	1	7	Probable
Tanaka S. 2017	2	2	0	0	0	0	1	5	Possible
Skopets I. 2015	2	2	0	0	-1	1	1	5	Possible
Bordin P 2015.	2	2	0	0	-1	0	1	4	Possible
Tham Y.L. 2002	2	2	2	0	-1	1	0	6	Probable

**Table 4:** Quality assessment of case report

Case ID	Selection	Ascertainment		Causality		Reporting	Total score	Quality assessment
	1. Does the patient(s) represent(s) the whole experience of the investigator (centre) or is the selection method unclear to the extent that other patients with similar presentation may not have been reported?	2. Was the exposure adequately ascertained?	3. Was the outcome adequately ascertained?	4. Were other alternative causes that may explain the observation ruled out?	5. Was follow-up long enough for outcomes to occur?	6. Is the case(s) described with sufficient details to allow other investigators to replicate the research or to allow practitioners make inferences related to their own practice?				
New case 2018	No	Yes	Yes	Yes	Yes	Yes	5	Good
Tanaka S. 2017	No	Yes	Yes	Yes	Yes	Yes	5	Good
Skopets I. 2015	No	Yes	Yes	No	Yes	Yes	4	Fair
Bordin P 2015.	Yes	Yes	Yes	No	Yes	Yes	5	Good
Tham Y.L. 2002	No	Yes	Yes	No	Yes	Yes	4	Fair

## Discussion

### Important findings

This systematic review analyzes five case reports, including a pharmacovigilance case from our hospital, to assess the potential relationship between TZB and IC. Despite variability in the clinical characteristics of the patients, common factors were identified, such as the absence of a history of heart failure and normal baseline cardiac function. Following TZB treatment, patients experienced a significant decline in cardiac function, leading to symptomatic heart failure. The time to IC onset varied among cases, and the causality assessment of the adverse reaction ranged from possible to probable.

### Assessment with the previous evidence

Several studies have identified the role of TZB in cardiac dysfunction. ^15^^-^^18^^)^ The reported incidence of cardiac toxicity attributed to this biologic is 2.6%. ^19^^)^ While many cases are reversible, there are rare instances where the cardiotoxicity is irreversible even after stopping TZB and providing optimal cardioprotective therapy. ^20^^,^^21^^)^ This IC can result in long-term heart failure, which impacts patient quality of life and survival outcomes. This irreversible IC can lead to chronic heart failure, significantly affecting patient quality of life and long-term survival. ^22^ Unlike reversible cardiotoxicity, which often improves with appropriate management, irreversible damage may result in a progressive decline in cardiac function, increasing morbidity, healthcare costs, and the need for advanced heart failure therapies, including transplantation or mechanical support.

The exact mechanisms behind TZB-induced IC are not fully understood, but they may involve direct myocardial injury, inflammation, endothelial dysfunction, or individual patient factors. Despite the effectiveness of TZB in treating HER2-positive cancers, this potentially life-threatening side effect highlights the need for clinicians and regulators to stay informed about the evolving safety profiles of medications to ensure monitoring and early detection to mitigate the risk of permanent heart damage.

A notable finding is that none of the patients had a prior history of heart failure, and all had normal baseline LVEF (≥50%), suggesting that the observed IC may have been directly related to TZB therapy. After treatment, the average lowest LVEF dropped significantly to 28% (SD ± 7.11), and all patients developed symptomatic grade III heart failure, as classified by the NYHA. These findings highlight the potential severity of cardiotoxicity following TZB, even in patients without prior cardiovascular conditions. The duration of TZB ranged from 4 to 24 months, with a median time of 10 months from the start of therapy to the onset of IC. This underscores the importance of a long-term monitoring for cardiotoxicity in patients with TZB, as the risk of IC may not be immediately apparent and can manifest after several months of treatment. The variability in the duration of treatment prior to the onset of IC may reflect individual patient factors or differences in the underlying mechanisms of TZB-induced cardiotoxicity.

In our SR, the causality assessment for the relationship between TZB and IC ranged from possible to probable, reflecting the complexities involved in establishing a direct cause-and-effect association. While a clear temporal association was observed between TZB administration and the onset of IC in all cases, the heterogeneous nature of the clinical presentations complicates the attribution of IC solely to TZB. It is important to recognize the potential role of other contributing factors, such as previous or concomitant use of other cardiotoxic agents, such as anthracyclines ^23^^)^ , other pre-existing comorbidities, and individual susceptibility to cardiotoxic agents. ^24^^,^^25^^)^

Prior use of anthracyclines may increase the risk of cardiotoxicity, although the evidence remains imprecise (OR 4.64, 95% CI 0.59-36.7) ^(^^21^^-^^22^. In our SR, one patient received anthracyclines previously and two patients concomitantly with TZB and developed cardiotoxicity within 5 months of starting TZB. In these cases, the potential synergistic effect of anthracyclines and TZB might explain the IC. A similar pattern was observed in our patient, as the event occurred six months after completing doxorubicin treatment. Given that anthracycline-induced cardiotoxicity can manifest within one year, ^26^ this possibility cannot be ruled out. Additionally, the presence of risk factors, such as a prior decrease in LVEF-though not below 50%-may have contributed to the outcome. Therefore, we conclude that irreversible damage induced by trastuzumab may develop in patients with additional risk factors.

Despite not being specifically linked to IC, two meta-analyses have identified pre-existing factors for heart dysfunction such as hypertension (OR 2.01, 95% CI 1.30-3.09), age ≥60 years (OR 2.03, 95% CI 1.38-3.00), a history of cardiovascular disease (OR 6.27, 95% CI 2.22-17.69), smoking (OR 1.33, 95% CI 1.07-1.65), BMI >23 (OR 2.96, 95% CI 1.18-7.37), and diabetes mellitus (OR 1.49, 95% CI 1.22-1.81). ^21^^,^^22^ In our SR, none of the patients had heart failure or abnormal LVEF values before starting TZB.

The median age was 52.6 years (SD ± 7.30), which may support the hypothesis that IC developed due to TZB alone or the synergistic effect of TZB and anthracyclines. However, age alone is not a definitive determinant of cardiotoxicity. While older age is considered a risk factor for cardiac dysfunction, it does not independently confirm TZB as the primary cause. Other factors, like obesity and diabetes mellitus, which are mentioned in two cases may have contributed to the development of IC, and it may explain the “possible” and “probable” relationship between TZB and IC.

Physician-related factors may also play a role in the development of IC, particularly in the challenges of early recognition. Our patient experienced respiratory difficulty after her twelfth cycle of TZB, and a multi-slice computed tomography scan of the thorax revealed interstitial pneumonitis (IP). Despite being afebrile and showing no significant changes in white blood cell count or bronchial secretion cultures, she was diagnosed with community-acquired pneumonia, although a viral process cannot be ruled out in an immunocompromised patient. The initial lack of suspicion for TZB-induced IP or cardiac dysfunction led to the continuation of TZB treatment, which may have contributed to the progression of long-term IC. It is important to note that IP is a rare reaction to TZB, occurring in approximately 0.5% of patients ^27^, and its potential role in the development of cardiotoxicity underscores the need for rigorous clinical monitoring.

### Limitations and strengthens

To our current understanding, this may be the first SR of specific cases of IC. Although we perform a global search, one limitation is the relatively small number of case reports identified, which limits the generalizability of the findings. Likewise, the heterogeneity in the clinical characteristics of the patients, such as comorbidities and use of concomitant therapies, may affect the interpretation of the results. The retrospective nature of the included case reports also introduces potential biases, such as incomplete data or underreporting of adverse events. Furthermore, the lack of long-term follow-up data in some cases limits our understanding of the full scope of the cardiotoxic effects of TZB. Additionally, some case reports lacked comprehensive clinical information, including baseline cardiac function, details on concomitant therapies, and standardized criteria for diagnosing cardiotoxicity, which may affect the interpretation of the findings. In our case, the absence of strain data from the initial echocardiograms, as well as the lack of serial NT-proBNP and troponin measurements, limits our ability to fully characterize early myocardial dysfunction and its progression over time. Finally, some case reports do not include a detailed assessment of this association.

Despite these limitations, this SR provides valuable insights into the potential for IC associated with TZB, particularly through its inclusion of a pharmacovigilance case from our hospital. This case adds a practical, real-world perspective to the existing literature. Moreover, by systematically analyzing case reports from diverse clinical settings, this review contributes to a broader understanding of the clinical presentation and progression of TZB-induced cardiotoxicity. The use of standardized causality assessment tools further strengthens the validity of the findings, allowing for a more consistent evaluation of the relationship between TZB and cardiac dysfunction.

In conclusion, while TZB is typically considered a reversible cardiotoxic agent, this SR highlights its potential risk for IC, with a median diagnosis time of 10 months and an average 30% reduction in LVEF. Though rare, IC can lead to severe heart failure, even in patients with no prior cardiovascular issues. The findings stress the need to consider other risk factors, such as concomitant therapies and comorbidities. Clinicians and regulators should be vigilant in monitoring and intervening early to mitigate long-term effects, and further research is needed to better understand the mechanisms and management of TZB-induced IC.

## Material suplementario

Irreversible cardiotoxicity induced by trastuzumab: a systematic review based on a pharmacovigilance case report

**Supplementary file 1:** SEARCH STRATEGY

Database	Search strategy	Results
PUBMED	((((trastuzumab[MeSH Terms]) OR (((trastuzumab[Title/Abstract]) OR (herceptin[ Title/Abstract])) OR (Trazimera[Title/Abstract])))) AND ((((((Breast Neoplasms[ MeSH Terms]) OR (Breast Tumor[Title/Abstract])) OR (Breast Cancer[Title/ Abstract])) OR (Mammary Cancer[Title/Abstract])) OR (“Cancer of Breast”[Title/ Abstract])))) AND (((((cardiotoxicity[MeSH Terms]) OR (Cardiac Toxicity[Title/ Abstract])) OR (cardiotoxicity[Title/Abstract])) OR (“cardiac dysfunction”[Title/ Abstract])) AND (((irreversi*) OR (“not reversi*”))))	16
SCOPUS	( TITLE-ABS-KEY ( “Breast Neoplasms” OR “Breast Tumor” OR “Breast Cancer” OR “Mammary Cancer” OR “cancer off breast” ) ) AND ( TITLE-ABS-KEY ( trastuzumab OR herceptin OR trimera ) ) AND ( ( TITLE-ABS-KEY ( cardiotoxicity OR “Cardiac Toxicity” OR “cardiac dysfunction” ) ) AND ( TITLE-ABS-KEY ( irreversible OR “not reversible” )))	49
WEB OF SCIENCE	TS=(Breast NEAR/3 Neoplasms) OR TS=(Breast NEAR/3 Cancer) OR TS=(Breast NEAR/3 Tumor) OR TS=(Mammary NEAR/3 Cancer) OR TS=(“Cancer of Breast”) AND ((TS=(trastuzumab )) OR TS=(herceptin )) OR TS=(trazimera ) AND TS=(irreversi* NEAR cardiotoxicity ) OR TS=(irreversi* NEAR “CARDIAC DYSFUNCTION”)	15
EMBASE	(“Breast tumor”:ab,ti OR “Breast Neoplasms”:ab,ti OR “Breast Cancer”:ab,ti OR “Mammary Cancer”:ab,ti OR “Cancer of Breast”:ab,ti ) AND (trastuzumab:ti,ab,tn OR herceptin:ti,ab OR Trazimera:ti,ab) AND (‘cardiotoxicity’:ti,ab OR ‘cardiac toxicity’:ti,ab OR ‘cardiac dysfunction’:ti,ab) AND (irreversi*:ti,ab)	45
